# Supplementary figures and images for: Lotus japonicus karrikin receptors display divergent ligand-binding specificities and organ-dependent redundancy
Source: PLoS Genet. 2020 Dec 28;16(12):e1009249. doi: 10.1371/journal.pgen.1009249 (PMC7808659; doi:10.1371/journal.pgen.1009249)

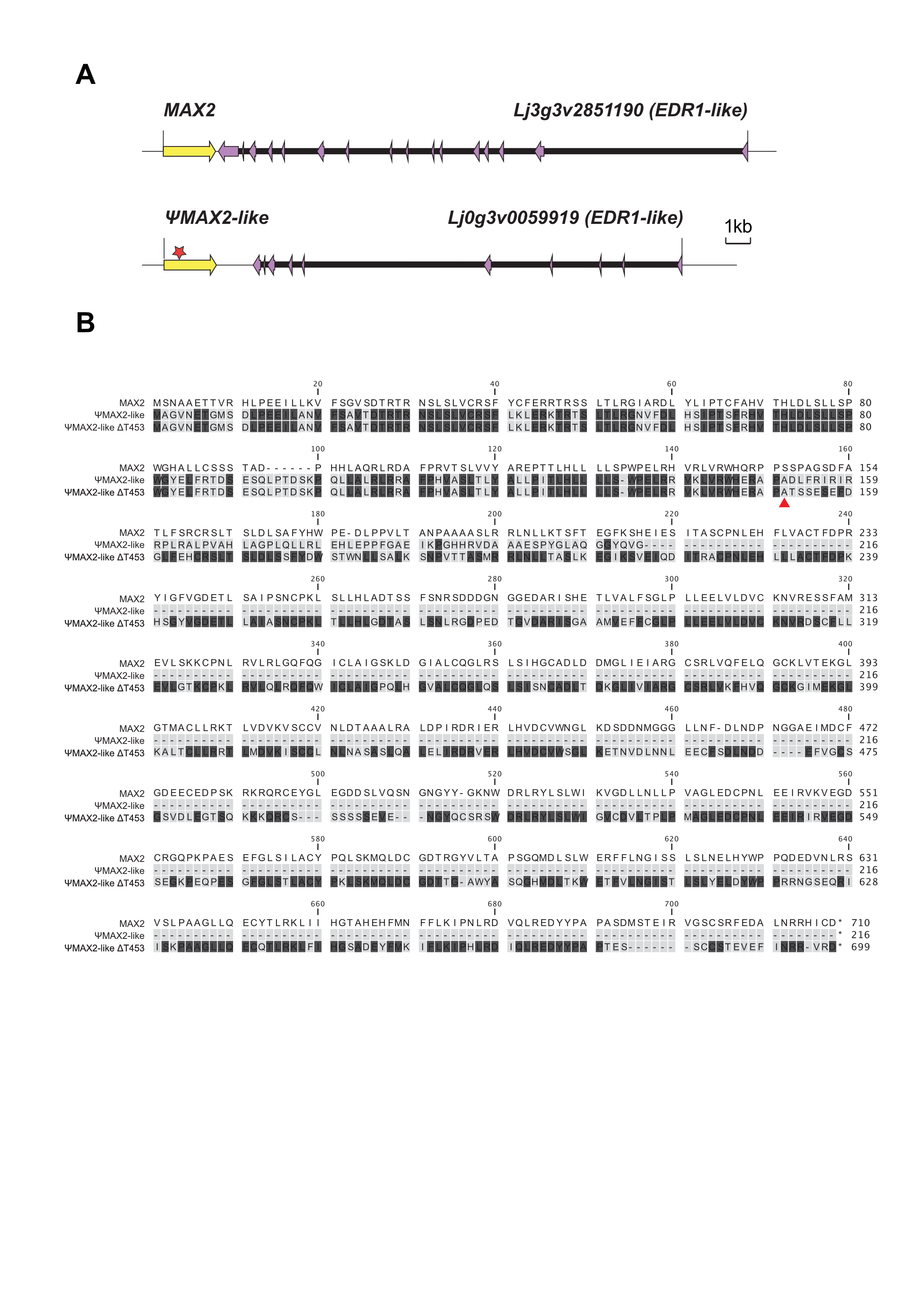

Supplement: S1 Fig — (A) Schematic representation of the syntenic regions containing the MAX2 and MAX2-like loci in L. japonicus. Coloured arrows and black lines show exons and introns respectively. (B) Protein alignment of LjMAX2, LjMAX2-like and an artificial LjMAX2-like with a deletion of the thymine at the position 453 in the coding sequence (LjMAX2-like ΔT453). Position of the nucleotide deletion is indicated in the translated sequence by a red triangle. Amino-acid conservation between MAX2 and MAX2-like is indicated by a dark background. (TIFF) [file pgen.1009249.s001.TIFF]

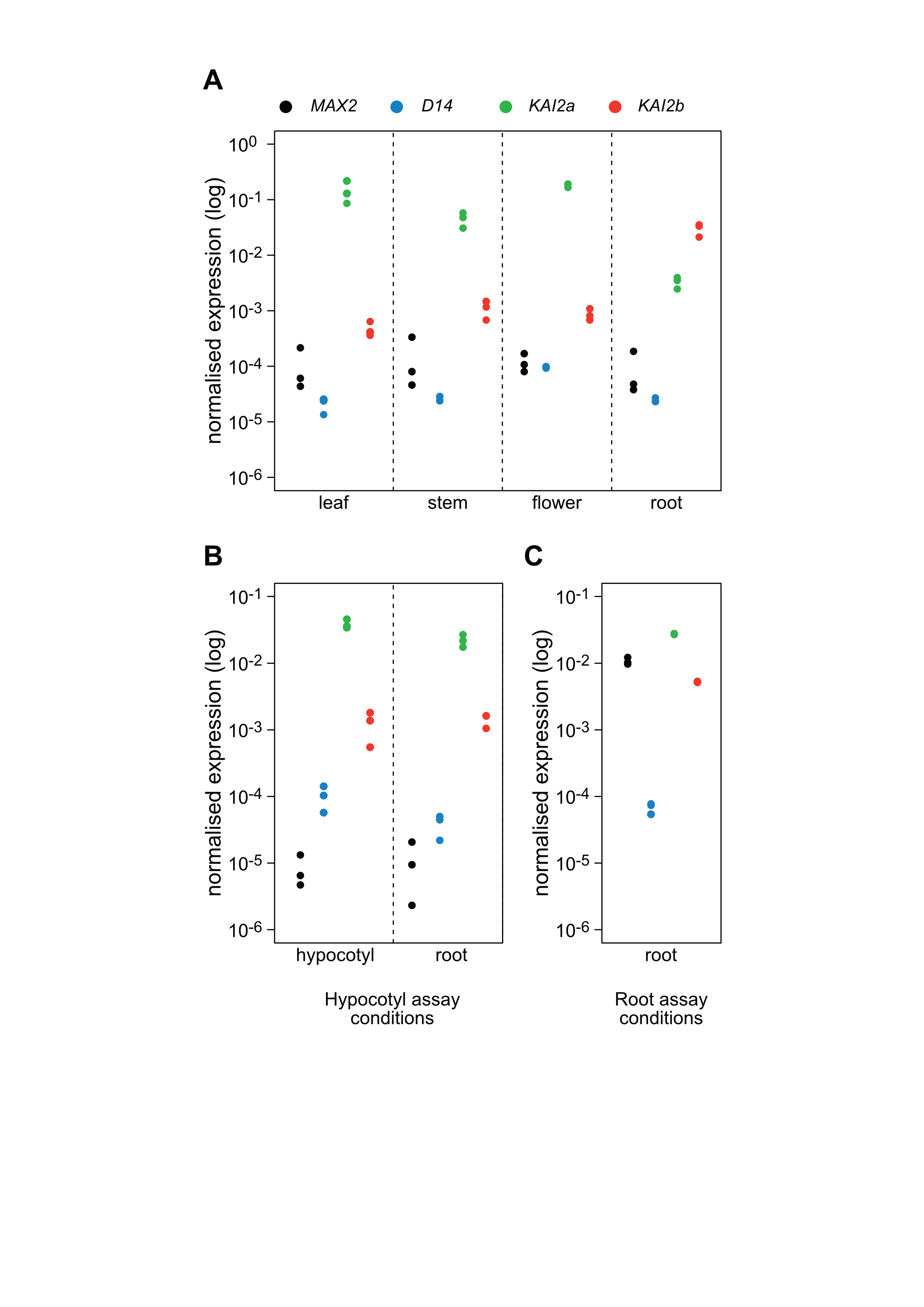

Supplement: S2 Fig — (A-C) Transcript accumulation in wild-type of D14, KAI2a, KAI2b and MAX2 normalized to expression of Ubiquitin, in (A) leaf, stem, flower and root of plants grown in pots, and in (B) hypocotyl and roots of 1 wpg plants grown on Petri dishes in 8h light /16h dark cycles, and in (c) roots of 2 wpg plants grown on Petri dishes in 16h light/8h dark cycles (n = 3). (TIFF) [file pgen.1009249.s002.tiff]

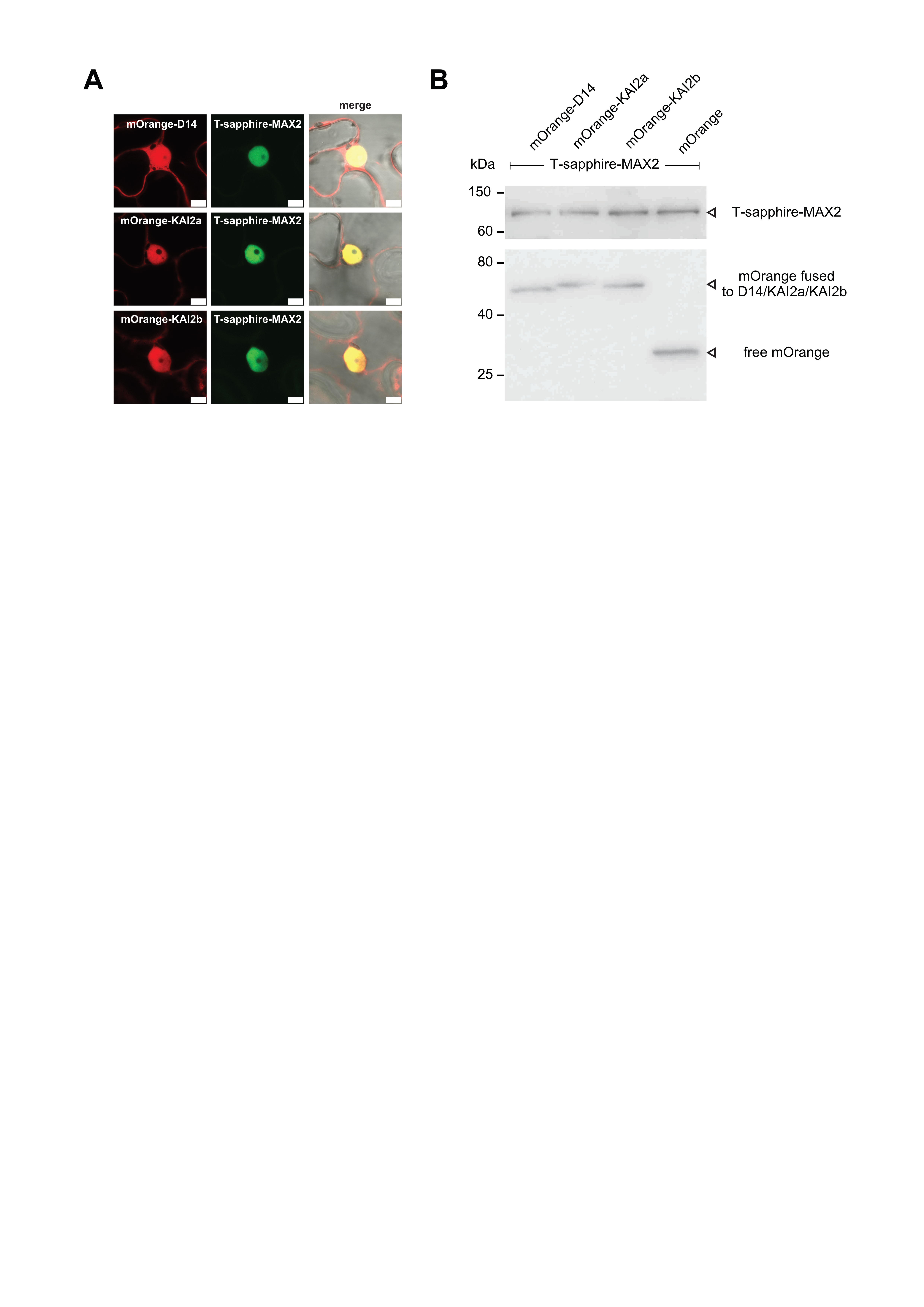

Supplement: S3 Fig — (A) Subcellular localization of LjD14, LjKAI2a, LjKAI2b and LjMAX2 in N. benthamiana leaf epidermal cells. LjD14, LjKAI2a and LjKAI2b are N-terminally fused with mOrange. LjMAX2 is N-terminally fused with T-Sapphire. Scale bars: 25 μm. (B) Western blot of protein extracts from N. benthamiana, showing that the mOrange tag fused with LjD14, LjKAI2a and LjKAI2b was not cleaved at detectable amounts. (TIFF) [file pgen.1009249.s003.TIFF]

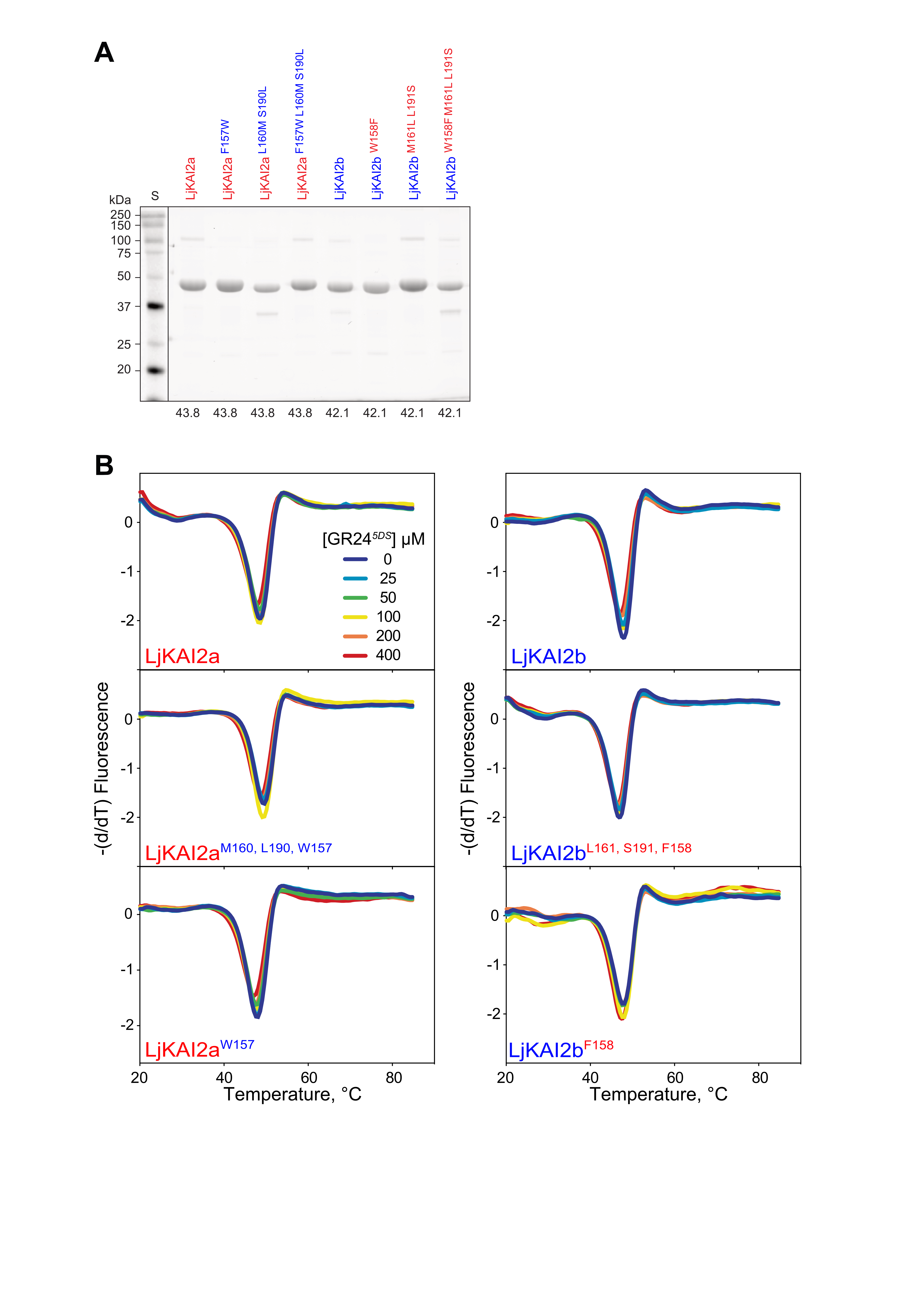

Supplement: S4 Fig — (A) 200 pmol (approx. 8 μg) of purified proteins were separated by 12% SDS-PAGE containing 2,2,2-trichlorethanol as a visualization agent. Below each lane is the calculated protein size in kilodaltons. S, protein size standards (Precision Plus Dual Color Standards, Bio-Rad #1610394) with corresponding sizes in kDa shown on the left. Optimal exposures of recombinant proteins and size standards were taken separately under UV transillumination and red epi-illumination, respectively. The two images were merged in post-processing, and the junction between them is indicated by a vertical line. (B) DSF curves of purified SUMO fusion proteins of wild-type LjKAI2a and LjKAI2b, and versions with swapped amino acids LjKAI2aW157,M160,L190, LjKAI2bF158,L161,S191, LjKAI2aW157, LjKAI2bF158, at the indicated concentrations of GR245DS. The first derivative of the change of fluorescence was plotted against the temperature. Each curve is the arithmetic mean of four technical replicates. Peaks indicate the protein melting temperature. There is no ligand-induced thermal destabilisation consistent with no protein-ligand interaction. (TIFF) [file pgen.1009249.s004.TIFF]

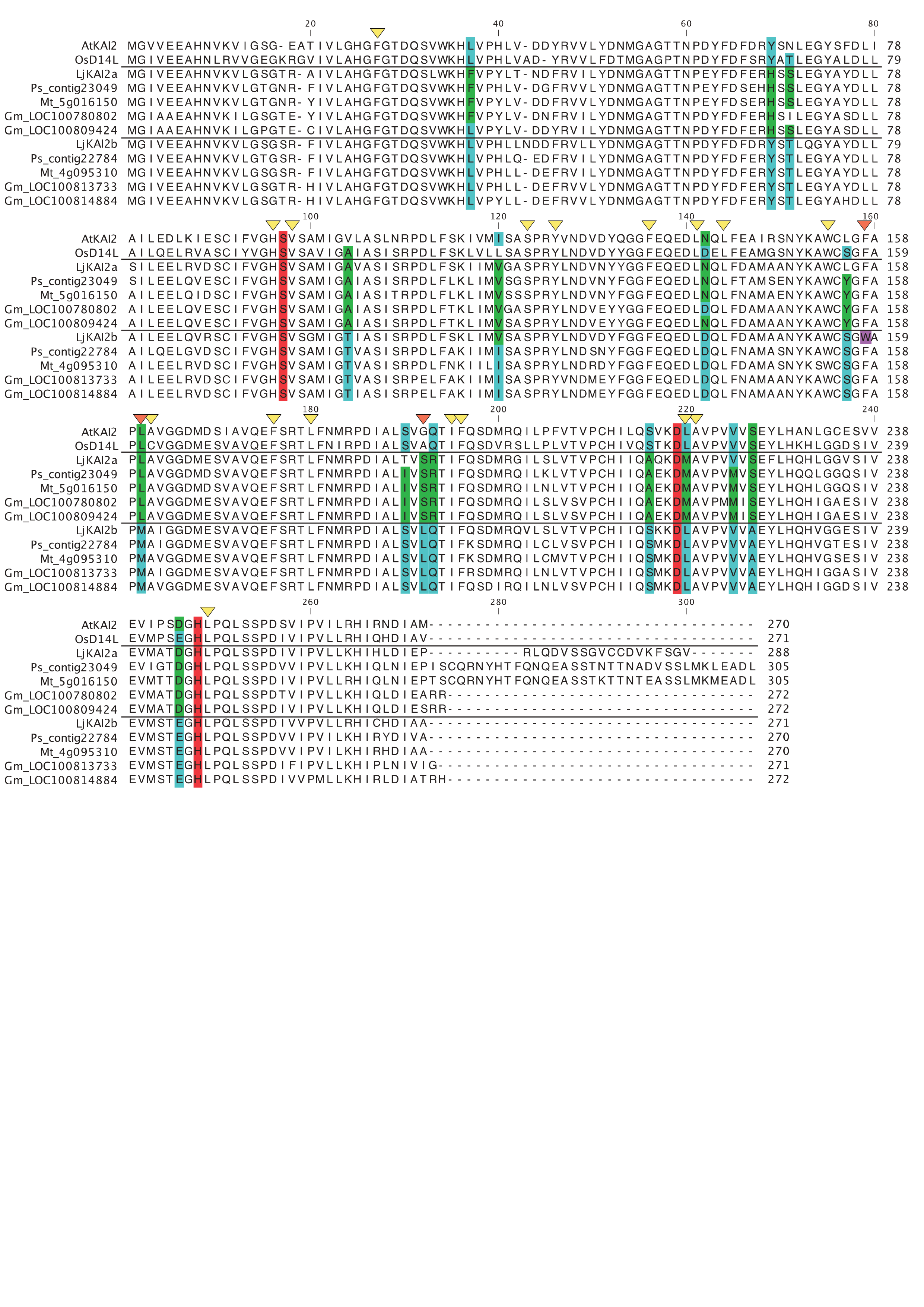

Supplement: S5 Fig — Protein sequence alignment of KAI2a and KAI2b homologs from the legumes Lotus japonicus, Pisum sativum, Medicago truncatula and Glycine max, in comparison with Arabidopsis KAI2 and rice D14L. Residues conserved within the KAI2a and KAI2b clades but different between these clades are coloured in green and blue. Residues of the catalytic triad are coloured in red. A non-conserved tryptophan in LjKAI2b located in the protein cavity is coloured in violet. Yellow triangles indicate amino acid residues located in the ligand-binding cavity of the proteins. Orange triangles indicate the three amino acids responsible for differences in GR24ent-5DS-binding between LjKAI2a and LjKAI2b. (TIFF) [file pgen.1009249.s005.TIFF]

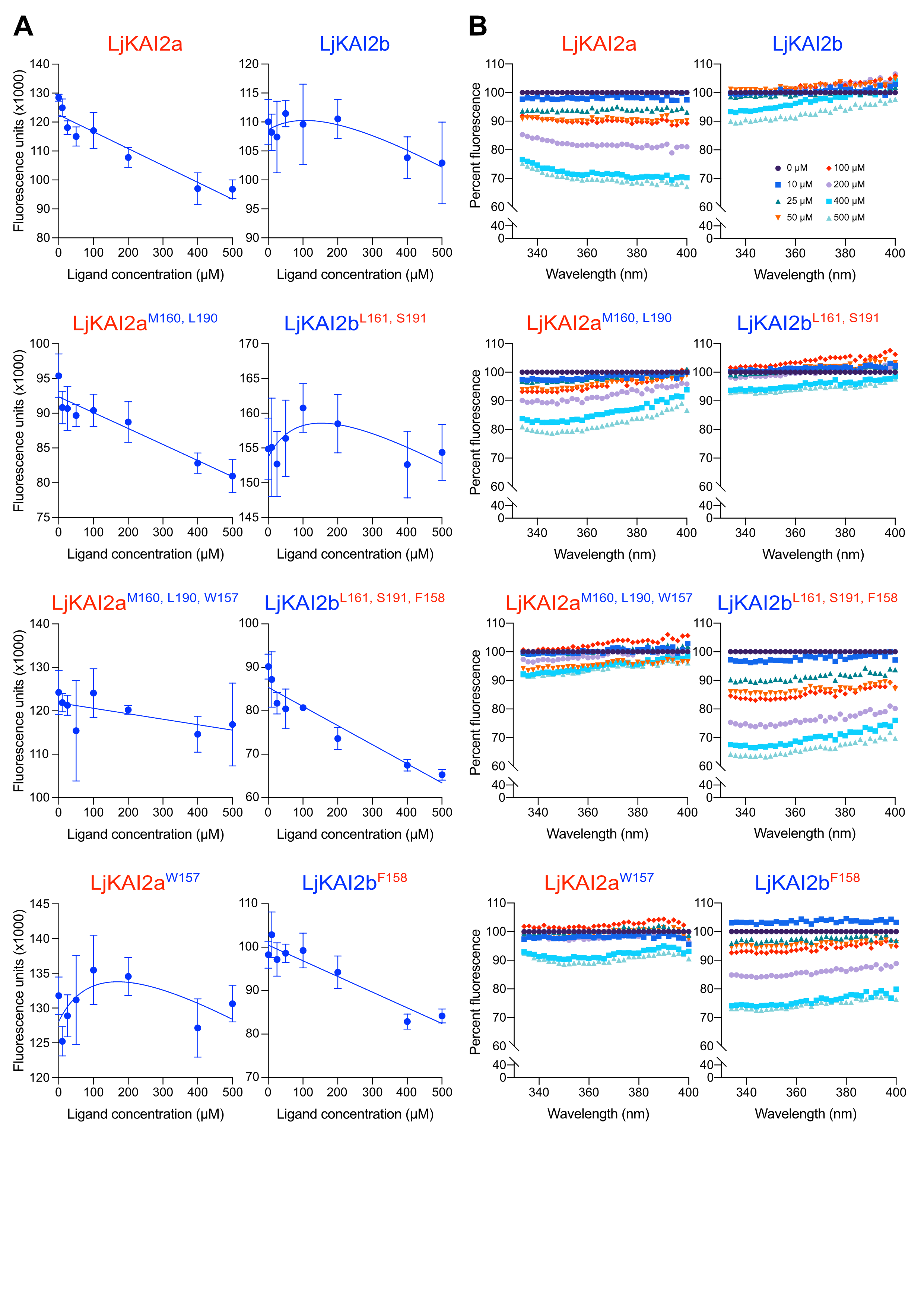

Supplement: S6 Fig — Intrinsic tryptophane fluorescence of wild-type LjKAI2a and LjKAI2b, and protein versions with swapped amino acids LjKAI2aM160,L190, LjKAI2bL161,S191, LjKAI2aM160,L190,W157, LjKAI2bL161,S191,F158, LjKAI2aW157, LjKAI2bF158 measured with (A) fixed wavelength filters (excitation 295/10 nm; longpass dichroic 325 nm; emission 360/20 nm) and (B) with a linear variable filter monochromator for emission wavelength scans (excitation 295/10 nm, emission 334–400 nm, step width 2 nm, emission bandwidth 8 nm) at the indicated GR24ent-5DS concentrations. (TIFF) [file pgen.1009249.s006.TIFF]

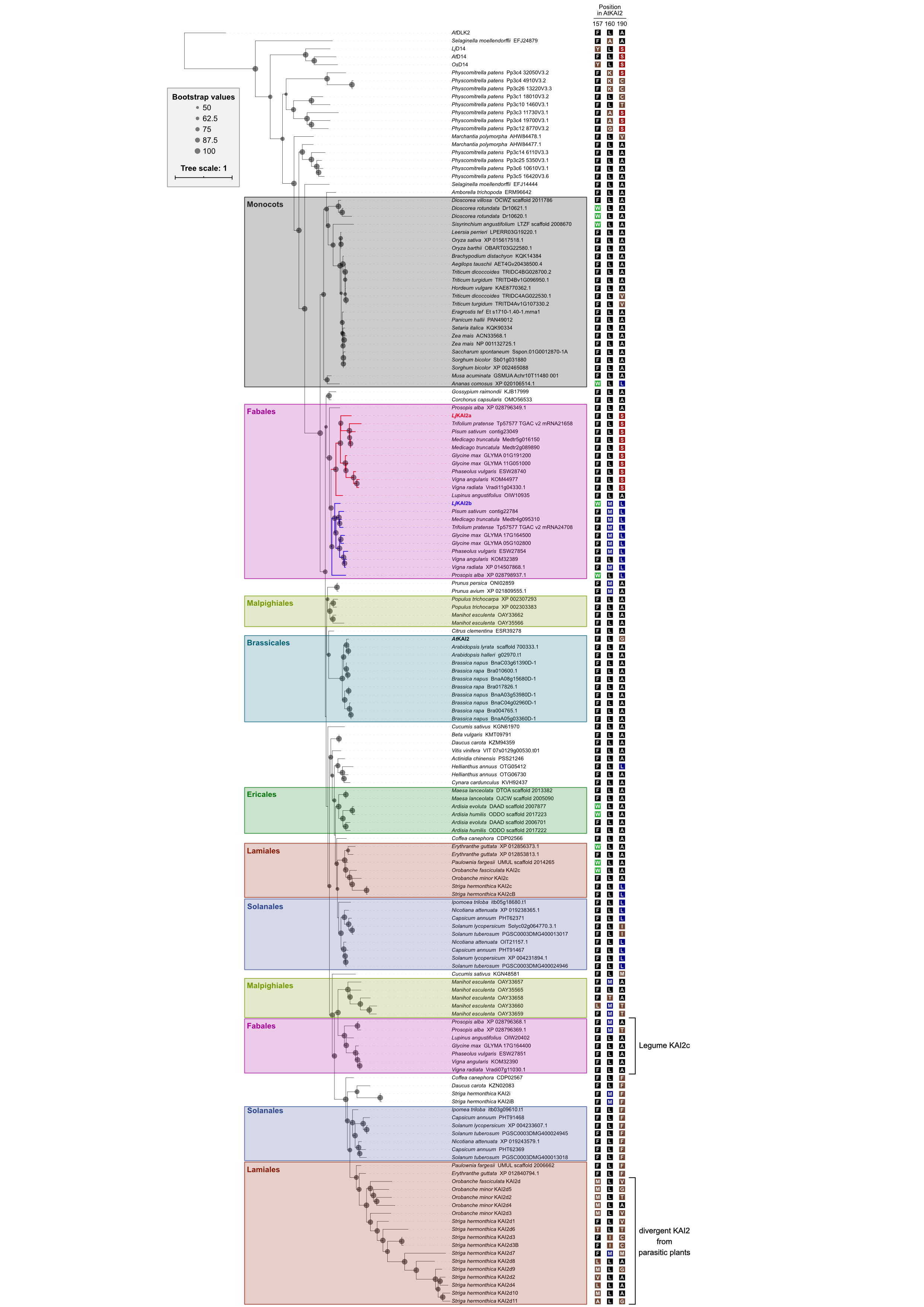

Supplement: S7 Fig — Phylogenetic tree of KAI2 proteins rooted with A. thaliana DLK2. The KAI2a and KAI2b clades in legumes are highlighted by red and blue branches. Monophyletic groups corresponding to a same order or clade are highlighted by coloured rectangular boxes. Amino-acids at the positions corresponding to AtKAI2 157, 160 and 190 are indicated with single-letter code. A black background indicates the presence of the most common residues in KAI2 proteins: F157, L160 and A190. A blue background indicates residues M160 and L190, conserved in legume KAI2b. A red background indicates S190, conserved in legume KAI2a. A green background indicates a W at position 157. A brown background indicates a different residue. (TIFF) [file pgen.1009249.s007.TIFF]

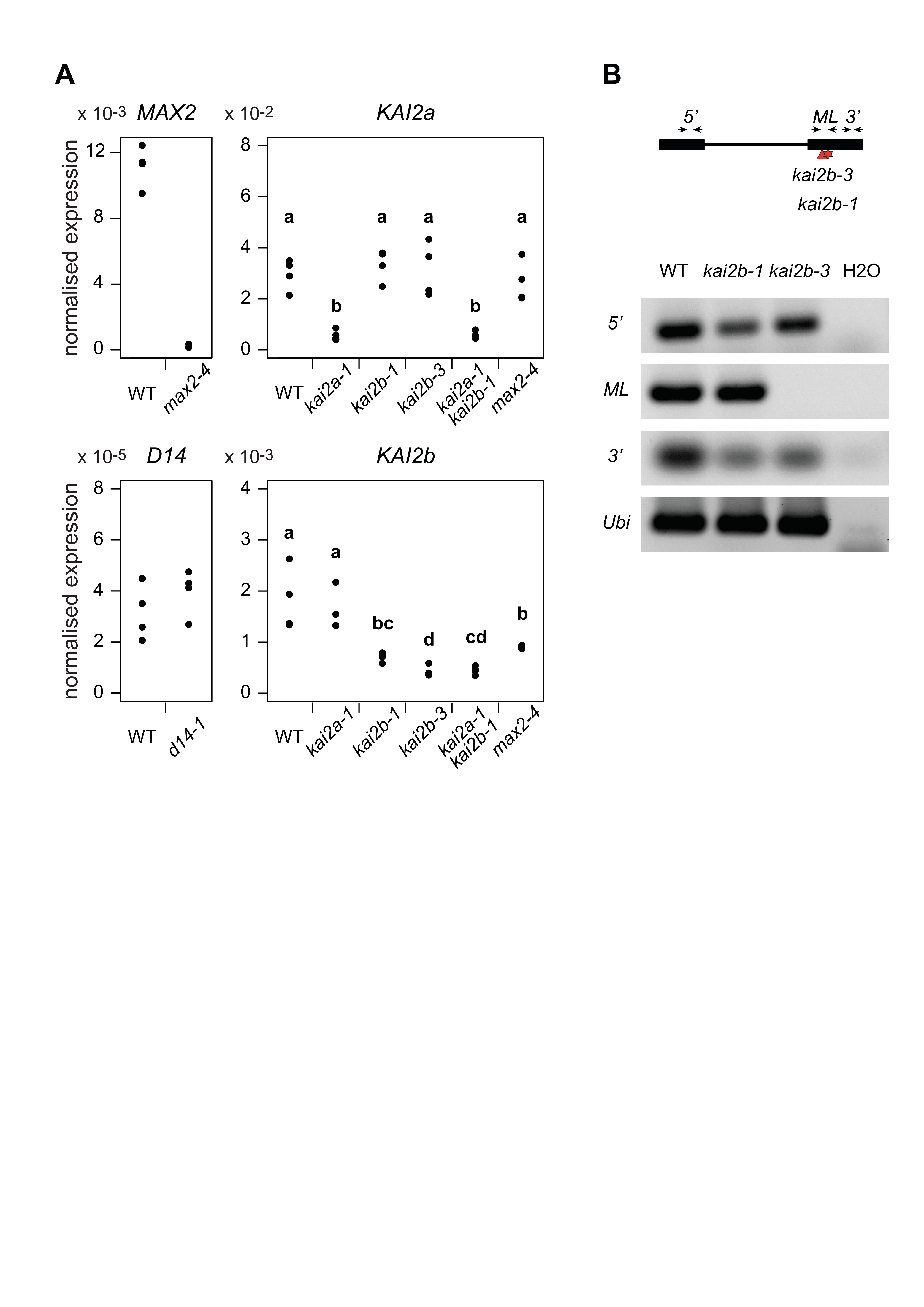

Supplement: S8 Fig — (A) qRT-PCR based transcript accumulation of LjKAI2a and LjKAI2b, in roots of wild type and kai2a-1, kai2b-1, kai2b-3, kai2a-1 kai2b-1 and max2-4 as well as LjMAX2 and LjD14 in max2-4 and d14-1, respectively (n = 4). Expression values were normalized to those of the housekeeping gene Ubiquitin. (B) LjKAI2b transcript accumulation in wild-type, kai2b-1 (stop codon) and kai2b-3 (LORE1 insertion) mutants by semi-quantitative RT-PCR using primer pairs located 5’ and 3’ of the mutations, as well as flanking (ML) the mutations. Transcript accumulation of the housekeeping gene Ubiquitin is also shown. (TIFF) [file pgen.1009249.s008.TIFF]

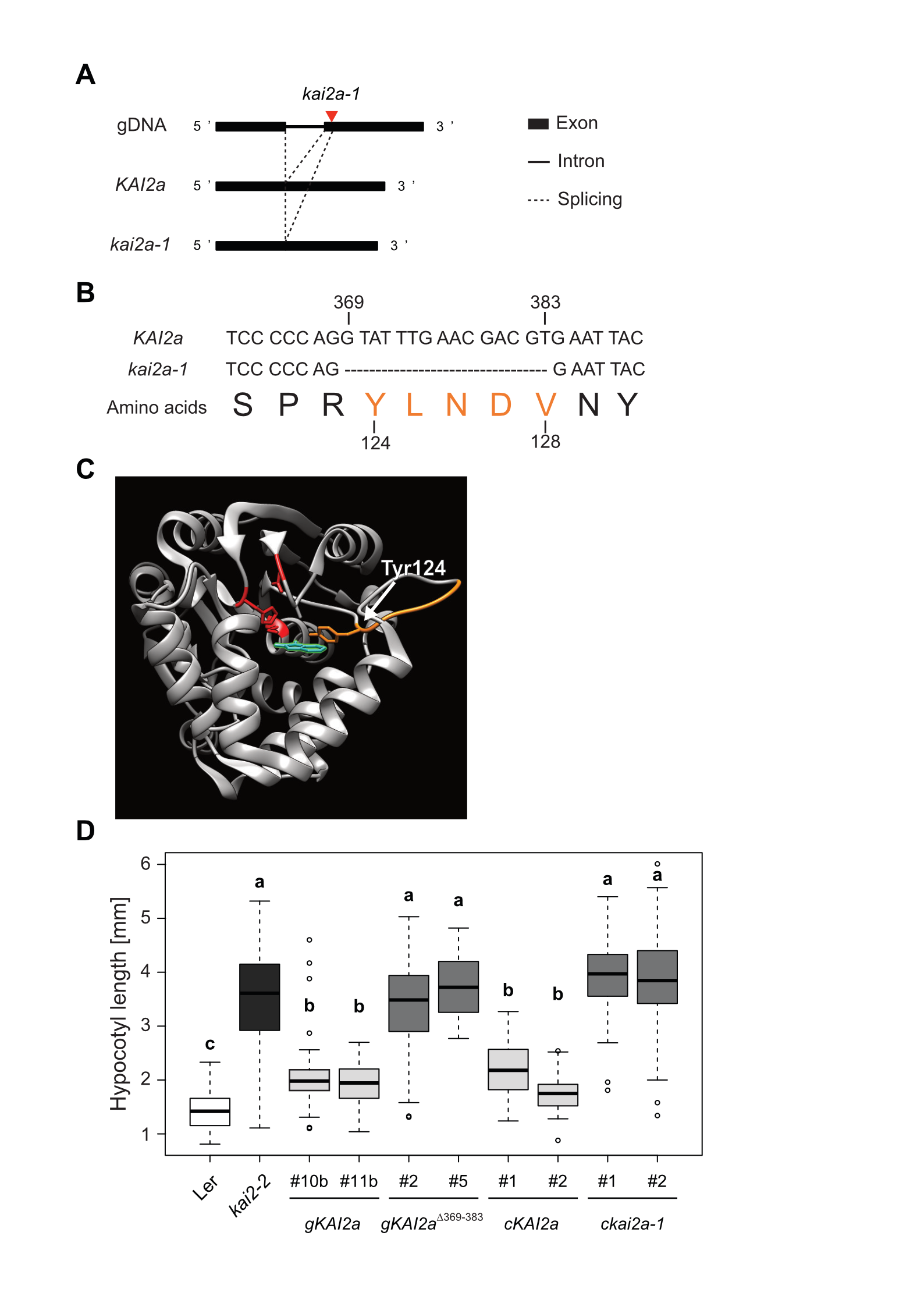

Supplement: S9 Fig — (A) Schematic representation of mis-splicing caused by the LORE1 insertion in the kai2a-1 mutant. (B) cDNA alignment showing the absence of nucleotides 369 to 383 in the kai2a-1 transcript, causing a deletion of amino acids 124 to 128 (orange). (C) Protein model of LjKAI2a based on the AtKAI2-KAR1 complex 4JYM [5] showing KAR1 in green, residues of the catalytic triad in red and the amino acids missing in a hypothetical LjKAI2a-1 protein in orange. (D) Hypocotyl elongation at 6 dpg in Arabidopsis kai2-2 mutants transgenically complemented with genomic and the cDNA of wild-type LjKAI2a and Ljkai2a-1 driven by the AtKAI2 promoter (n = 75–106). Plants were grown in 8h light/16h dark cycles. Letters indicate different statistical groups (ANOVA, post-hoc Tukey test). (TIFF) [file pgen.1009249.s009.TIFF]

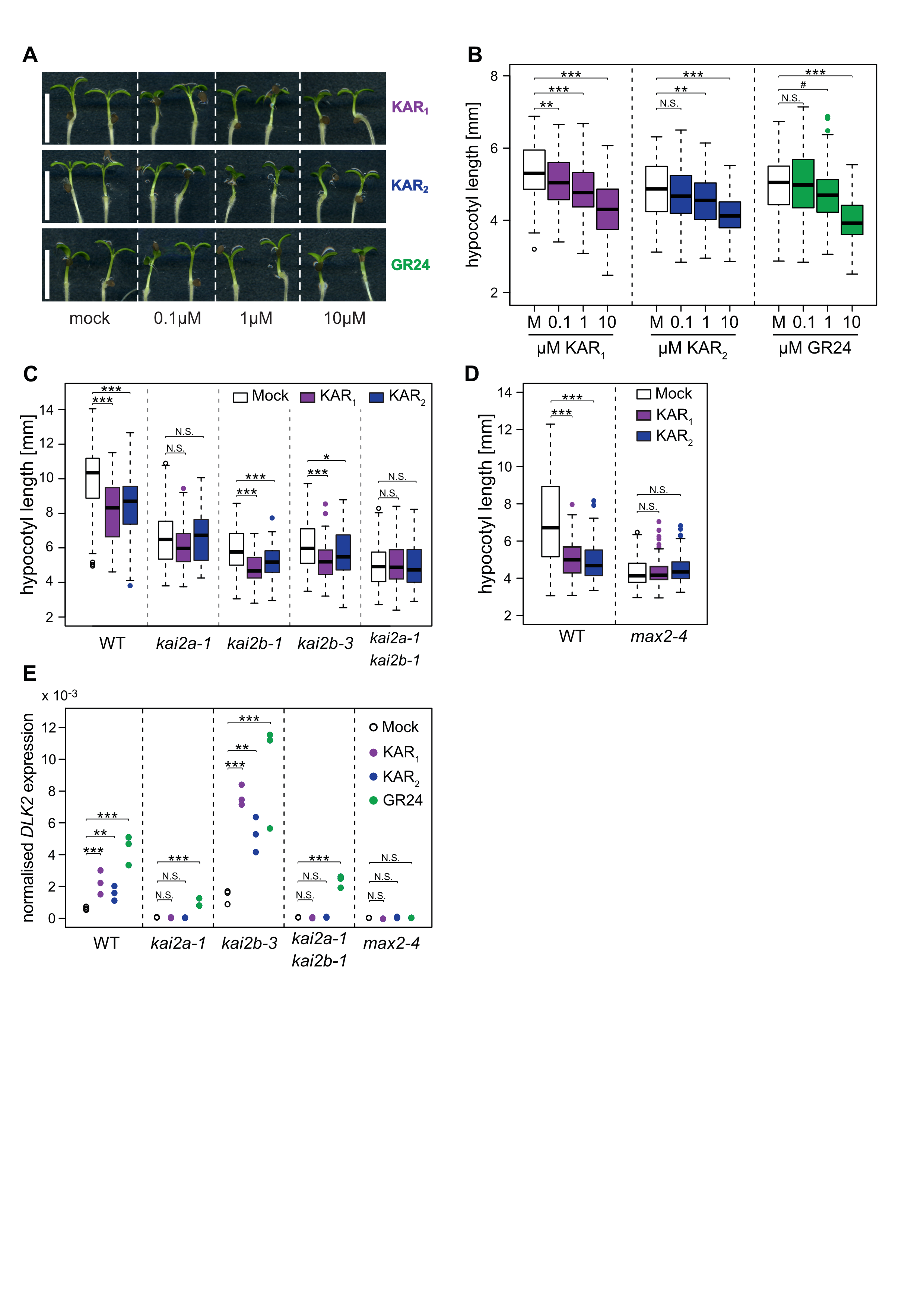

Supplement: S10 Fig — (A) Hypocotyls and (B) hypocotyl length of L. japonicus wild-type seedling at 1 wpg after treatment with solvent (M) or three different concentrations of KAR1, KAR2 or rac-GR24 (GR24) (n = 95–105). Letters indicate different statistical groups (ANOVA, post-hoc Tukey test). (C) Hypocotyl length of the indicated genotypes at 1 wpg after treatment with solvent (Mock), 1 μM KAR1 or 1 μM KAR2 (n = 73–107). (D) Hypocotyl length of wild-type and max2-4 seedlings 1 wpg after treatment with solvent (Mock), 1 μM KAR1, 1 μM KAR2 (n = 66–96). (E) RT-qPCR-based expression of DLK2 in hypocotyls at 1 wpg after 2 hours treatment with solvent (Mock), 1 μM KAR1, 1 μM KAR2, or 1 μM rac-GR24 (GR24) (n = 3). Expression values were normalized to those of the housekeeping gene Ubiquitin. (A-E) Seedlings were grown in 8h light/16h dark cycles. (C-E) Asterisks indicate significant differences of the compounds versus mock treatment (ANOVA, post-hoc Dunnett test, N.S.>0.05, *≤0.05, **≤0.01, ***≤0.001). (TIFF) [file pgen.1009249.s010.TIFF]

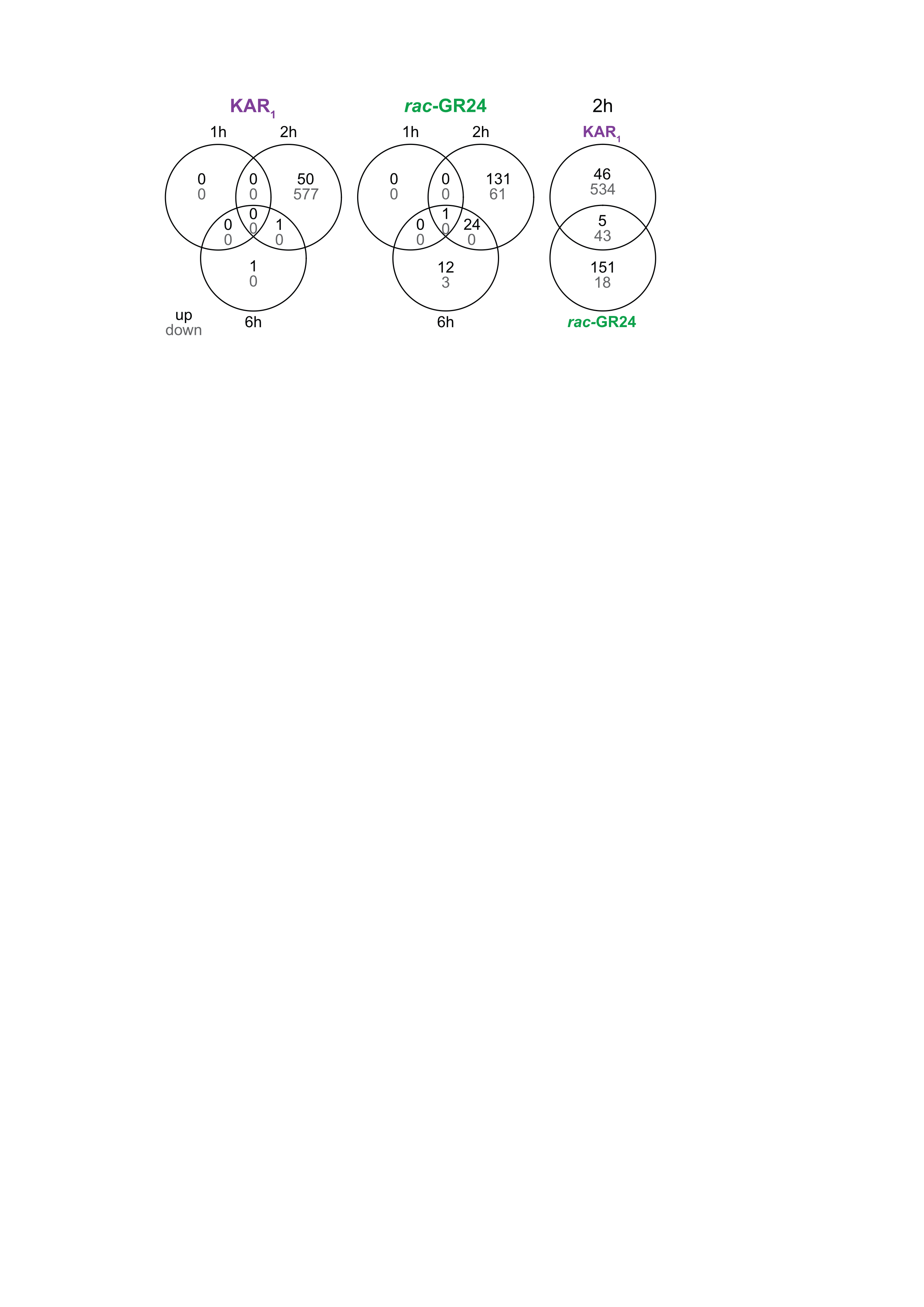

Supplement: S11 Fig — Number of differentially expressed genes (DEGs, adjusted p-value < 0.01) as assessed by microarray analysis. Left panel: DEGs responding to 1 μM KAR1 after 1h, 2h and 6h incubation. Middle panel: DE genes responding to 1 μM rac-GR24 1h, 2, 6h incubation. Right panel: comparison of DE genes responding to 2 h treatment with KAR1 and rac-GR24. (TIFF) [file pgen.1009249.s011.TIFF]

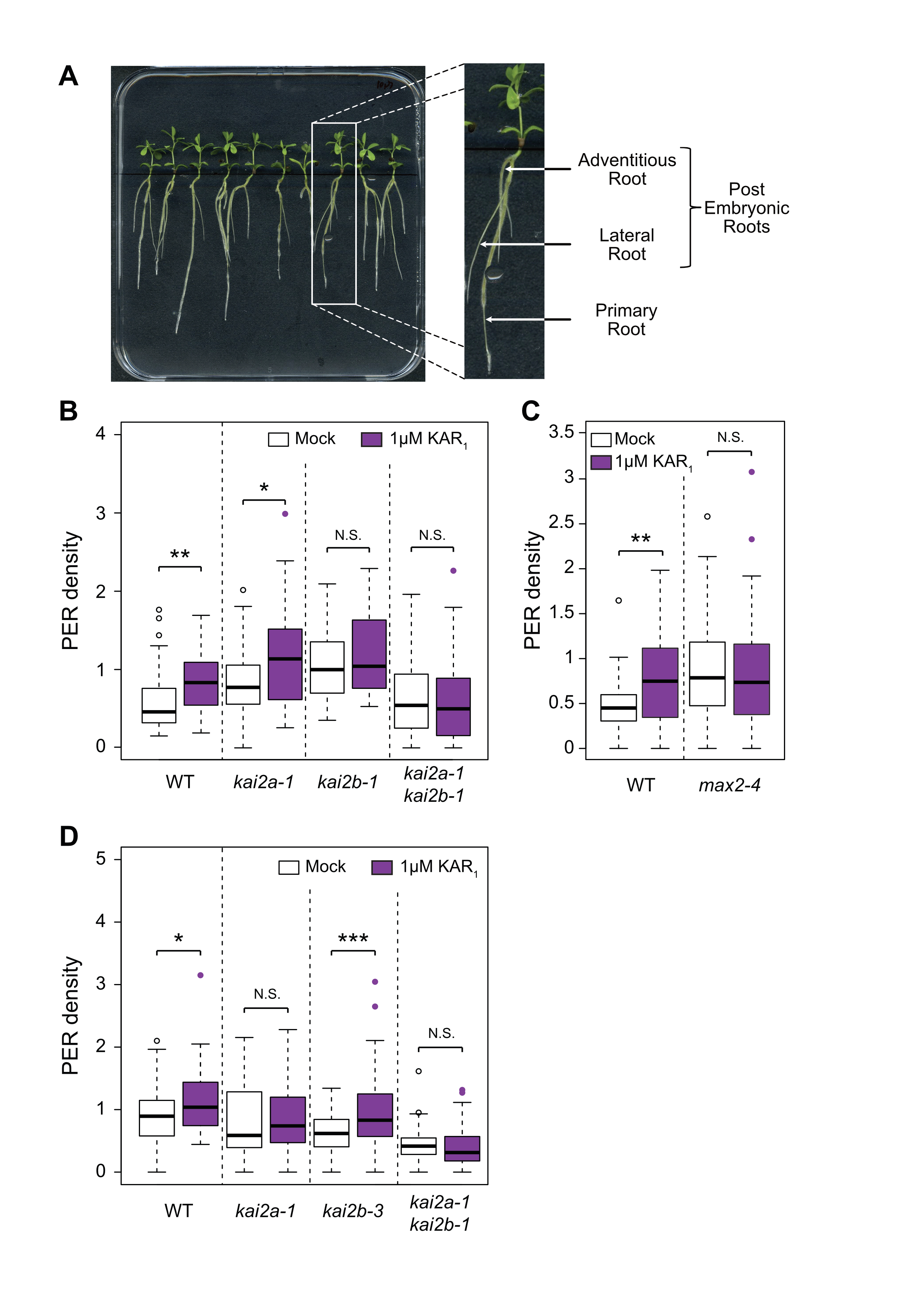

Supplement: S12 Fig — (A) Image of Lotus japonicus seedling with indicated post-embryonic roots (PERs). (B-D) Post-embryonic-root (PER) density of L. japonicus plants, 2 wpg after treatment with solvent (Mock) or 1 μM KAR1, of wild-type, (B) kai2a-1, kai2b-1 and kai2a-1 kai2b-1 (n = 32–50); (C) max2-4 (n = 34–43); (D) kai2a-1, kai2b-3 and kai2a-1 kai2b-1 (n = 37–72). (B-D) Asterisks indicate significant differences versus mock treatment (Welch t.test, *≤0.05, **≤0.01, ***≤0.001). (TIFF) [file pgen.1009249.s012.TIFF]

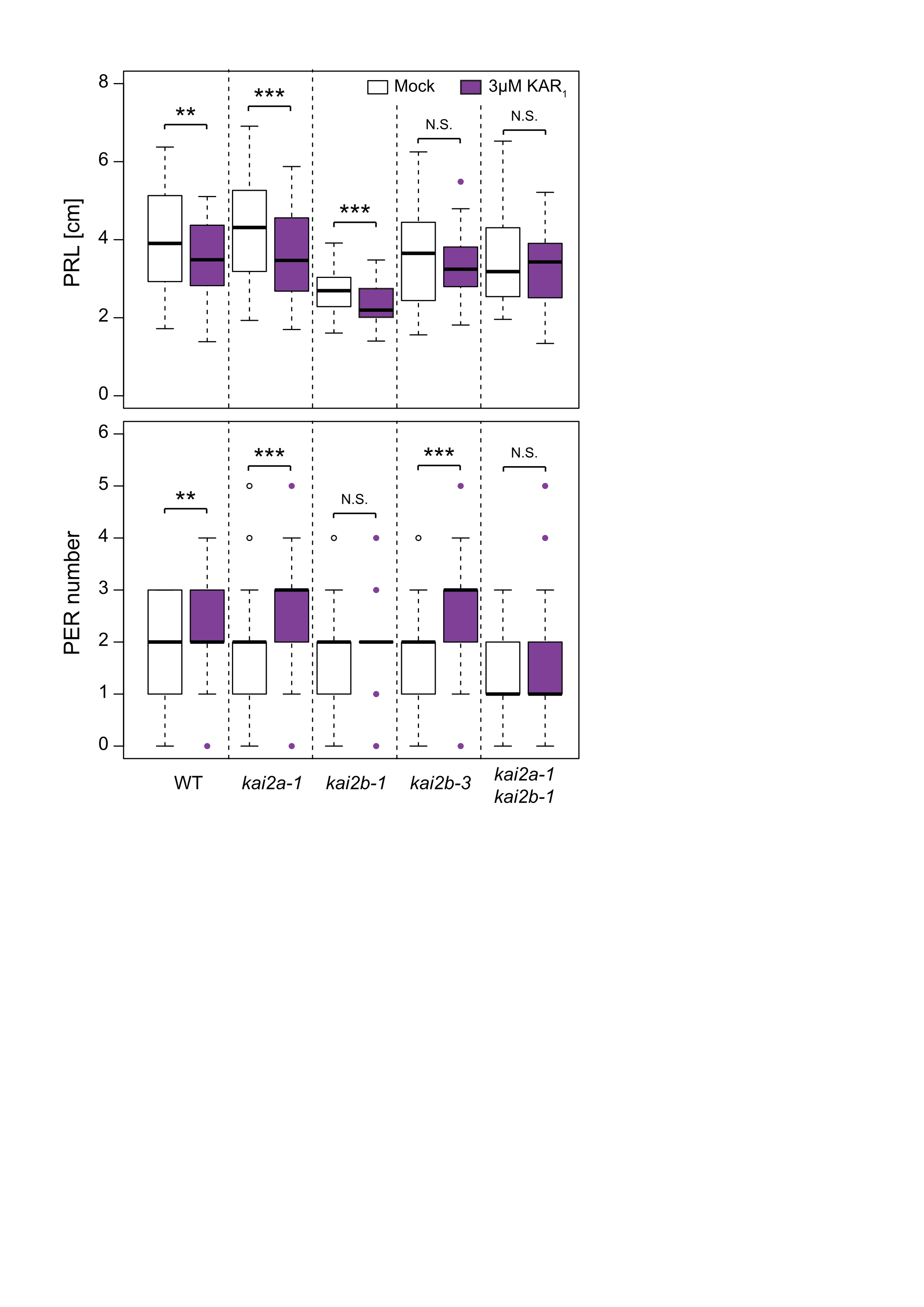

Supplement: S13 Fig — Primary-root length (PRL) and post-embryonic-root (PER) number of L. japonicus plants, 2 wpg after treatment with solvent (Mock) or 3 μM KAR1 (n = 34–72) displayed in Fig 9A. Asterisks indicate significant differences versus mock treatment (Welch t.test, *≤0.05, **≤0.01, ***≤0.001). (TIFF) [file pgen.1009249.s013.TIFF]
