# Supplementary material for: Lotus japonicus karrikin receptors display divergent ligand-binding specificities and organ-dependent redundancy
Source: PLoS Genet. 2020 Dec 28;16(12):e1009249. doi: 10.1371/journal.pgen.1009249 (PMC7808659; doi:10.1371/journal.pgen.1009249)
Supplement: S1 Table — (DOCX) [file pgen.1009249.s014.docx]

**S1 Table.** *L. japonicus* mutants used in this study and information on seed production.

| **allele** | **type** | **reference** | **position from ATG** | **comments** |
| --- | --- | --- | --- | --- |
| *Ljd14-1* | EMS | SL4580 | C685T (Q > stop) | hardly produce flowers |
| *Ljkai2a-1* | LORE1 insertion | 30008990 | 387 | - |
| *Ljkai2b-1* | EMS | SL1281 | C640T (Q > stop) | - |
| *Ljkai2b-2* | EMS | SL2723 | G462A (W > stop) | produced no seeds |
| *Ljkai2b-3* | LORE1 insertion | 30034333 | 535 | - |
| *Ljmax2-1* | LORE1 insertion | 30031159 | 83 | hardly produce flowers |
| *Ljmax2-2* | LORE1 insertion | P0860_3 | 504 | hardly produce flowers |
| *Ljmax2-3* | LORE1 insertion | 30019601 | 1132 | produce few flowers |
| *Ljmax2-4* | LORE1 insertion | 30049531 | 1230 | produce few flowers |
