# Supplementary material for: Lotus japonicus karrikin receptors display divergent ligand-binding specificities and organ-dependent redundancy
Source: PLoS Genet. 2020 Dec 28;16(12):e1009249. doi: 10.1371/journal.pgen.1009249 (PMC7808659; doi:10.1371/journal.pgen.1009249)
Supplement: S3 Table — (DOCX) [file pgen.1009249.s016.docx]

**S3 Table.** Primers

**Primers used for LORE1 insertion mutant genotyping.** Forward primer was used to amplify specifically LORE1 insertion with a specific P2 primer (CCATGGCGGTTCCGTGAATCTTAGG).

| **mutant** | **Forward** | | **Reverse** | |
| --- | --- | --- | --- | --- |
| *Ljkai2a-1* | Sc403 | TATGGTCTCTCACGCTGTTTCCGCC  ATGATCG | Sc283 | TCCACAATAGACACGCCACC |
| *Ljkai2b-3* | Sc285 | CCTCCGTTGACATGACCTCC | Sc17 | TTGAAGACTACCCCTTAAACA  AGGGGTTTGAG |
| *Ljmax2-1* | Sc130 | ATGAAGACTTTACGGGTCTCACACC  ATGAGTAACGCTGCTGAAAC | CG416 | CAGTAGAAGCTCCGGCAAAC |
| *Ljmax2-2* | CG383 | TTGGGGAGGGGTTTAATAGG | CG424 | CGATTTCGTGAGACTTGAAGC |
| *Ljmax2-3* | Sc163 | TCACCTCGCTGGATCTCTC | Sc131 | TTGAAGACTACCACCTCCCAT  GTTGTCATC |
| *Ljmax2-4* | Sc131 | TTGAAGACTACCACCTCCCATGTTG  TCATC | Sc163 | TCACCTCGCTGGATCTCTC |

**Primers used for EMS mutant genotyping.** dCAPS strategy was used to genotype EMS mutants.

| **mutant** | **Forward** | | **Reverse** | | **Site** |
| --- | --- | --- | --- | --- | --- |
| *Ljd14-1* | Sc429 | GCCGGCGGCGGCCGCGAGGT  ACCTG | Sc242 | TTTCGTCTCACCTTGTGTGCCC  CCGCCAGTGC | PstI  (Cut WT) |
| *Ljkai2b-1* | Sc431 | GGTAACTGTGCCATGTCACAG  TATA | Sc285 | CCTCCGTTGACATGACCTCC | AccI  (Cut WT) |

**Primers used for cloning.**

| **Use** | **Primers** | |
| --- | --- | --- |
| cloning promoter *AtD14* in LI | Sc224 | TTTCGTCTCAGCGGGTCTACACATTCATCAATCTCGC |
|  | Sc225 | TTTCGTCTCACAGATTTTTTATGTGTTTGGGTTTGAG |
| cloning promoter *AtKAI2* fragment 1 in LI | Sc232 | TTTCGTCTCAGCGGGGCGATTCAGTGCCATGATT |
|  | Sc233 | TTTCGTCTCACGATTCGTTCAGATTCTCGCT |
| cloning promoter *AtKAI2* fragment 2 in LI | Sc234 | TTTCGTCTCAATCGACTCGAATTTGATGGATCTTTC |
|  | Sc235 | TTTCGTCTCACAGACTCTCTAAAGAAGATTCTTC |
| cloning genomic *AtD14* in LI | Sc236 | TTTCGTCTCACACCATGAGTCAACACAACATCTTAGAAG |
|  | Sc237 | TTTCGTCTCACCTTTCACCGAGGAAGAGCTCGCC |
| cloning genomic *AtKAI2* in LI | Sc238 | TTTCGTCTCACACCATGGGTGTGGTAGAAGAAG |
|  | Sc239 | TTTCGTCTCACCTTTCACATAGCAATGTCATTACGAATG |
| cloning genomic *LjD14* in LI | Sc240 | TTTCGTCTCACACCATGGCCACTTCAATCCTCGACG |
|  | Sc241 | TTTCGTCTCACCTTTCAGTGTGCCCCCGCCAGTG |
| cloning genomic *LjKAI2a* and cDNA *Ljkai2a-1* in LI | Sc243 | TTTCGTCTCACACCATGGGGATAGTGGAGGAAGCTCAC |
|  | Sc244 | TTTCGTCTCACCTTTTACACCCCACTAAATTTTACATCAC |
| cloning genomic *LjKAI2b* in LI | Sc246 | TTTCGTCTCACACCATGGGGATAGTGGAAGAAGCTC |
|  | Sc247 | TTTCGTCTCACCTTTCAAGCTGCAATATCATGGCAAATG |
| cloning genomic *Ljkai2a-1* in LI | Sc243 | TTTCGTCTCACACCATGGGGATAGTGGAGGAAGCTCAC |
|  | ST97 | CAAATCCTTCCATAGTAATTTGCGGAAGAAAATCATC |
|  | ST96 | TTGAAGACTATCTTCAGATATCTCATATAC |
|  | Sc244 | TTTCGTCTCACCTTTTACACCCCACTAAATTTTACATCAC |
| cloning cDNA *LjKAI2a^M160,L190,W157^*  fragment 1 in L0 | Sc505 | ATGAAGACTTCCATCGGAGCCCACCCTAAAC |
|  | ST161 | ATGAAGACTTTACGTCGTCTCACACCATGGG |
| cloning cDNA *LjKAI2a^M160,L190,W157^*  fragment 2 in L0 | ST163 | ATGAAGACTTATGGCGGTGGGTGGAGACATG |
|  | ST164 | ATGAAGACTTCGCAAAACGGTTAGAGCAATATC |
| cloning cDNA *LjKAI2a^M160,L190,W157^*  fragment 3 in L0 | ST165 | ATGAAGACTTTGCGGACCATTTTTCAGAGC |
|  | Sc498 | ATGAAGACTACAGACGTCTCACCTTTTACACCCCACTAA  ATTTTAC |
| cloning cDNA *LjKAI2b ^L161,S191,F158^*  fragment 1 in L0 | Sc506 | ATGAAGACTTCCAGCGGGGCAAAGCCTGAAC |
|  | ST169 | ATGAAGACTTTACGTCGTCTCACACCATGGG |
| cloning cDNA *LjKAI2b ^L161,S191,F158^*  fragment 2 in L0 | ST171 | ATGAAGACTTCTGGCTATCGGAGGAGACATG |
|  | ST172 | ATGAAGACTTTGCGATACGCTTAAGGCTATG |
| cloning cDNA *LjKAI2b ^L161,S191,F158^*  fragment 3 in L0 | ST173 | ATGAAGACTTCGCAGACAATTTTTCAAAGTG |
|  | Sc503 | ATGAAGACTACAGACGTCTCACCTTTCAAGCTGCAATATC |
| cloning pSUMO *LjKAI2a^M160,L190^* | Sc604 | CGTGGTGTTTAGGGTTTGCTCCGATGGCGGTG |
|  | Sc605 | CACCGCCATCGGAGCAAACCCTAAACACCACG |
| cloning pSUMO *LjKAI2b ^L161,S191^* | Sc606 | CATGGTGTTCAGGCTGGGCCCCGCTGGCTATC |
|  | Sc607 | GATAGCCAGCGGGGCCCAGCCTGAACACCATG |
| cloning pSUMO *LjKAI2a^W157^* | ST177 | GTGGTGTTTAGGGTGGGCTC |
|  | ST178 | AGCGGAGCCCACCCTAAAC |
| cloning pSUMO *LjKAI2b ^F158^* | ST179 | ATGGTGTTCAGGCTTTGC |
|  | ST180 | ATAGCCATCGGGGCAAAG |
| cloning pENTR/D-TOPO *LjD14* | CG389 | CACCATGGCCACTTCAATCCTCGAC |
|  | CG390 | TCAGTGTGCCCCCGCCAG |
| cloning pENTR/D-TOPO *LjKAI2a* | CG385 | CACCATGGGGATAGTGGAGGAAGC |
|  | CG386 | TTACACCCCACTAAATTTTACATCA |
| cloning pENTR/D-TOPO *LjKAI2b* | CG387 | CACCATGGGGATATGTGGAAGAAGC |
|  | CG388 | TCAAGCTGCAATATCATGGC |
| cloning pENTR/D-TOPO *LjMAX2* | CG391 | CACCATGAGTAACGCTGCTGAAAC |
|  | CG392 | TCAATCACAGATATGACGCC |

**Primers used for gene amplification by RT-qPCR.**

| **Use** | **Primers** | |
| --- | --- | --- |
| qPCR *Ubiquitin* | Ubi F | ATGCAGATCTTCGTCAAGACCTTG |
|  | Ubi R | ACCTCCCCTCAGACGAAG |
| qPCR *LjMAX2* | Sc302 | GAATGTTACACCCTGAGGAAGC |
|  | Sc303 | TCAGGTTTGGGATCTTGAGG |
| qPCR *LjKAI2a* | Sc282 | CGGTGCAGGAGTTTAGCAGA |
|  | Sc283 | TCCACAATAGACACGCCACC |
| qPCR *LjKAI2b* | Sc284 | AAGAAAGACCTGGCGGTTCC |
|  | Sc285 | CCTCCGTTGACATGACCTCC |
| qPCR *LjDLK2* | MG027 | CTCCTTGGTGCTTCTCCCAG |
|  | MG028 | AAAGCCGAAGCCAGTTTTCA |
| qPCR *LjD14* | D14_qPCR_F | ACAGCGTCCGAGAAAACTC |
|  | D14_qPCR_R | AGCAATGGAGGCCAACTAC |
