# Supplementary material for: Lotus japonicus karrikin receptors display divergent ligand-binding specificities and organ-dependent redundancy
Source: PLoS Genet. 2020 Dec 28;16(12):e1009249. doi: 10.1371/journal.pgen.1009249 (PMC7808659; doi:10.1371/journal.pgen.1009249)
Supplement: S4 Table — (DOCX) [file pgen.1009249.s017.docx]

**S4 Table.** Plasmids

| **Name** | **Description** |
| --- | --- |
| **Golden Gate Level 0** | |
| L0 c*LjKAI2a^M160, L190,W157^* A | PCR amplification of *L. japonicus* Gifu cDNA with primers Sc505 +ST161. Assembly by StuI cut ligation into L0-Amp (BB01) |
| L0 c*LjKAI2a^M160, L190,W157^* B | PCR amplification of *L. japonicus* Gifu cDNA with primers ST163 +ST164. Assembly by StuI cut ligation into L0-Amp (BB01) |
| L0 c*LjKAI2a^M160, L190,W157^* C | PCR amplification of *L. japonicus* Gifu cDNA with primers ST165 +Sc498. Assembly by StuI cut ligation into L0-Amp (BB01) |
| L0 c*LjKAI2b ^L161,S191,F158^* A | PCR amplification of *L. japonicus* Gifu cDNA with primers Sc506 +ST169. Assembly by StuI cut ligation into L0-Amp (BB01) |
| L0 c*LjKAI2b ^L161,S191,F158^* B | PCR amplification of *L. japonicus* Gifu cDNA with primers ST171 +ST172. Assembly by StuI cut ligation into L0-Amp (BB01) |
| L0 c*LjKAI2b ^L161,S191,F158^* C | PCR amplification of *L. japonicus* Gifu cDNA with primers ST173 +Sc503. Assembly by StuI cut ligation into L0-Amp (BB01) |
| **Golden Gate Level I** | |
| LI Esp3I p*AtKAI2* A | PCR amplification of *L. japonicus* Gifu genomic DNA with primers Sc232 + Sc233. Assembly by StuI cut ligation into LI-pUC57 (BB02) |
| LI Esp3I p*AtKAI2 B* | PCR amplification of *L. japonicus* Gifu genomic DNA with primers Sc234 + Sc235. Assembly by StuI cut ligation into LI-pUC57 (BB02) |
| LI Esp3I p*AtD14* | PCR amplification of *L. japonicus* Gifu genomic DNA with primers Sc224 + Sc225. Assembly by StuI cut ligation into LI-pUC57 (BB02) |
| LI Esp3I *gAtKAI2* | PCR amplification of *L. japonicus* Gifu genomic DNA with primers Sc238 + Sc239. Assembly by StuI cut ligation into LI-pUC57 (BB02) |
| LI Esp3I *gAtD14* | PCR amplification of *L. japonicus* Gifu genomic DNA with primers Sc237 + Sc238. Assembly by StuI cut ligation into LI-pUC57 (BB02) |
| LI Esp3I *gLjKAI2a* | PCR amplification of *L. japonicus* Gifu genomic DNA with primers Sc243 + Sc244. Assembly by StuI cut ligation into LI-pUC57 (BB02) |
| LI Esp3I *gLjKAI2b* | PCR amplification of *L. japonicus* Gifu genomic DNA with primers Sc246 + Sc247. Assembly by StuI cut ligation into LI-pUC57 (BB02) |
| LI Esp3I *gLjD14* | PCR amplification of *L. japonicus* Gifu genomic DNA with primers Sc240 + Sc241. Assembly by StuI cut ligation into LI-pUC57 (BB02) |
| LI Esp3I *gLjkai2a-1* | PCR amplification of *L. japonicus* *kai2a-1* genomic DNA with primers Sc243 + ST97 and ST96 +Sc244. Assembly by Bpil and StuI cut ligation into LI-pUC57 (BB02) |
| LI Esp3I *cLjkai2a-1* | PCR amplification of *L. japonicus* *kai2a-1* coding DNA with primers Sc243 + Sc244. Assembly by StuI cut ligation into LI-pUC57 (BB02) |
| LI Esp3I *cLjKAI2a* | PCR amplification of *L. japonicus* Gifu coding DNA with primers Sc243 + Sc244. Assembly by StuI cut ligation into LI-pUC57 (BB02) |
| LI Esp3I c*LjKAI2b* | PCR amplification of *L. japonicus* Gifu cDNA with primers Sc246 + Sc248. Assembly by StuI cut ligation into LI-pUC57 (BB02) |
| LI Esp3I  c*LjKAI2a^M160, L190,W157^* | Assembled by BpiI cut ligation from: L0 c*LjKAI2a^M160, L190,W157^* A + L0 c*LjKAI2a^M160, L190,W157^* B + L0 c*LjKAI2a^M160, L190,W157^* C + LI-BpiI (BB03) |
| LI Esp3I  c*LjKAI2b ^L161,S191,F158^* | Assembled by BpiI cut ligation from: L0 c*LjKAI2b ^L161,S191,F158^* A + L0 c*LjKAI2b ^L161,S191,F158^* B + L0 c*LjKAI2b ^L161,S191,F158^* C + LI-BpiI (BB03) |
| **Golden Gate Level II** | |
| LIIc F 1-2 POI:GOI:*HygroR* | Assembled by BsaI cut ligation from: LI A-B POI (G082) + LI B-C dy (BB06) + LI C-D GOI + LI D-E dy (BB08) + LI E-F nos-T (G006) + LI F-G HygroR (G095) + LIIc F 1-2 (BB30) |
| LIIc R 3-4 p*35S:mCherry* | Assembled by BsaI cut ligation from: LI A-B p*35S* (G005) + LI B-C dy (BB06) + LI C-D *mCherry* (G023) + LI D-E dy (BB08) + LI E-F 35S-T (G059) + LI F-G dy (BB09) + LIIc R 3-4 (BB34) |
| **Golden Gate Level III** | |
| LIIIβ POI:GOI:*HygroR* | Assembled by BpiI cut ligation from: LIIc F 1-2 POI:GOI:HygroR + LII 2-3 ins (BB43) + LIIc R 3-4 p35S*:mCherry* + LII 4-6 dy (BB41) + LIIIβ F A-B (BB53) |
| LIIIβ p*AtKAI2:gAtKAI2* | Assembled by Esp3I cut ligation from: LIIIβ F A-B POI:GOI:HygroR + LI Esp3I *pAtKAI2* A + LI Esp3I *pAtKAI2* A + LI Esp3I *gAtKAI2* |
| LIIIβ p*AtKAI2:gAtD14* | Assembled by Esp3I cut ligation from: LIIIβ F A-B POI:GOI:HygroR + LI Esp3I *pAtKAI2* A + LI Esp3I *pAtKAI2* A + LI Esp3I *gAtD14* |
| LIIIβ p*AtKAI2:gLjKAI2a* | Assembled by Esp3I cut ligation from: LIIIβ F A-B POI:GOI:HygroR + LI Esp3I *pAtKAI2* A + LI Esp3I *pAtKAI2* A + LI Esp3I *gLjKAI2a* |
| LIIIβ p*AtKAI2:gLjKAI2b* | Assembled by Esp3I cut ligation from: LIIIβ F A-B POI:GOI:HygroR + LI Esp3I *pAtKAI2* A + LI Esp3I *pAtKAI2* A + LI Esp3I *gLjKAI2b* |
| LIIIβ p*AtKAI2: gLjkai2a-1* | Assembled by Esp3I cut ligation from: LIIIβ F A-B POI:GOI:HygroR + LI Esp3I *pAtKAI2* A + LI Esp3I *pAtKAI2* A + LI Esp3I *gLjkai2a-1* |
| LIIIβ p*AtKAI2: cLjkai2a-1* | Assembled by Esp3I cut ligation from: LIIIβ F A-B POI:GOI:HygroR + LI Esp3I *pAtKAI2* A + LI Esp3I *pAtKAI2* A + LI Esp3I *cLjkai2a-1* |
| LIIIβ p*AtKAI2:gLjD14* | Assembled by Esp3I cut ligation from: LIIIβ F A-B POI:GOI:HygroR + LI Esp3I *pAtKAI2* A + LI Esp3I *pAtKAI2* A + LI Esp3I *gLjD14* |
| LIIIβ p*AtD14:gAtD14* | Assembled by Esp3I cut ligation from: LIIIβ F A-B POI:GOI:HygroR + LI Esp3I *pAtKD14*  + LI Esp3I *gAtD14* |
| LIIIβ p*AtD14:gAtKAI2* | Assembled by Esp3I cut ligation from: LIIIβ F A-B POI:GOI:HygroR + LI Esp3I *pAtKD14*  + LI Esp3I *gAtKAI2* |
| LIIIβ p*AtD14:gLjD14* | Assembled by Esp3I cut ligation from: LIIIβ F A-B POI:GOI:HygroR + LI Esp3I *pAtKD14*  + LI Esp3I *gLjD14* |
| LIIIβ p*AtD14:gLjKAI2a* | Assembled by Esp3I cut ligation from: LIIIβ F A-B POI:GOI:HygroR + LI Esp3I *pAtKD14*  + LI Esp3I *gLjKAI2a* |
| LIIIβ p*AtD14:gLjKAI2b* | Assembled by Esp3I cut ligation from: LIIIβ F A-B POI:GOI:HygroR + LI Esp3I *pAtKD14*  + LI Esp3I *gLjKAI2b* |
| **Protein induction** | |
| pSUMO c*LjKAI2a* | PCR amplification from LI Esp3I *cLjKAI2a* with primers MW1002 + MW1003. Assembly by Gibson cloning |
| pSUMO c*LjKAI2b* | PCR amplification from LI Esp3I *cLjKAI2b* with primers MW1002 + MW1004. Assembly by Gibson cloning |
| pSUMO  c*LjKAI2a^M160, L190,W157^* | PCR amplification from LI Esp3I *cLjKAI2a (3b)* with primers MW1002 + MW1003. Assembly by Gibson cloning |
| pSUMO  c*LjKAI2b ^L161,S191,F158^* | PCR amplification from LI Esp3I *cLjKAI2b (3a)* with primers MW1002 + MW1004. Assembly by Gibson cloning |
| pSUMO  c*LjKAI2a^M160, L190^* | Rolling circle PCR amplification from pSUMO *LjKAI2a (3b)* with primers Sc604 + Sc605. |
| pSUMO  c*LjKAI2b ^L161,S191,F158^* | Rolling circle PCR amplification from pSUMO *LjKAI2b (3a)* with primers Sc606 + Sc607. |
| pSUMO c*LjKAI2^,W157^* | Rolling circle PCR amplification from pSUMO *LjKAI2a* with primers ST177 + ST178. |
| pSUMO c*LjKAI2b^,F158^* | Rolling circle PCR amplification from pSUMO *LjKAI2b* with primers ST179 + ST180. |
| **Protein localization** | |
| pENTR/D-TOPO *LjD14* | PCR amplification of *L. japonicus* Gifu genomic DNA with primers CG389 + CG390. Assembly by directional cloning in pENTR/D-TOPO. |
| pENTR/D-TOPO *LjKAI2a* | PCR amplification of *L. japonicus* Gifu genomic DNA with primers CG385 + CG386. Assembly by directional cloning in pENTR/D-TOPO. |
| pENTR/D-TOPO *LjKAI2b* | PCR amplification of *L. japonicus* Gifu genomic DNA with primers CG387 + CG388. Assembly by directional cloning in pENTR/D-TOPO. |
| pENTR/D-TOPO *LjMAX2* | PCR amplification of *L. japonicus* Gifu genomic DNA with primers CG391 + CG392. Assembly by directional cloning in pENTR/D-TOPO. |
| *p35s:mOrange-LjD14* | Cloned by LR reaction with pENTR/D-TOPO *LjD14* in *35s:mOrange-GW-Nos*. |
| *p35s:mOrange-LjKAI2a* | Cloned by LR reaction with pENTR/D-TOPO *LjKAI2a* in *35s:mOrange-GW-Nos*. |
| *p35s:mOrange-LjKAI2b* | Cloned by LR reaction with pENTR/D-TOPO *LjKAI2b* in *35s:mOrange-GW-Nos*. |
| *p35s:mOrange-LjMAX2* | Cloned by LR reaction with pENTR/D-TOPO *LjMAX2* in *35s:TSaphire-GW-Nos*. |
