# Supplementary material for: Lotus japonicus karrikin receptors display divergent ligand-binding specificities and organ-dependent redundancy
Source: PLoS Genet. 2020 Dec 28;16(12):e1009249. doi: 10.1371/journal.pgen.1009249 (PMC7808659; doi:10.1371/journal.pgen.1009249)
Supplement: S5 Table — (DOCX) [file pgen.1009249.s018.docx]

**S5 Table.** Results of ANOVA for multiple comparisons.

| **Figure** | **genotype/treatment/gene** | ***post hoc* test** | **p-value** | **F-value** |
| --- | --- | --- | --- | --- |
| Fig. 2A | - | Tukey | ≤ 0.001 | F_14/1438_ = 125.3 |
| Fig. 2C | - | Tukey | ≤ 0.001 | F_11/132_ = 45.6 |
| Fig. 3B | WT (Ler) | Tukey | ≤ 0.001 | F_2/311_ = 244 |
|  | *kai2-2* |  | = 0.18 | F_2/300_ = 1.71 |
|  | *AtKAI2 #1* |  | ≤ 0.001 | F_2/122_ = 31.9 |
|  | *AtKAI2 #3* |  | ≤ 0.001 | F_2/303_ = 116.4 |
|  | *LjKAI2a #10b* |  | ≤ 0.001 | F_2/316_ = 65.7 |
|  | *LjKAI2a #11b* |  | ≤ 0.001 | F_2/313_ = 42 |
|  | *LjKAI2b #1b* |  | ≤ 0.001 | F_2/296_ = 33.4 |
|  | *LjKAI2b #5b* |  | ≤ 0.001 | F_2/288_ = 87.4 |
| Fig. 3C | WT (Col) | Tukey | ≤ 0.001 | F_2/311_ = 158.3 |
|  | K02821 |  | ≤ 0.001 | F_2/353_ = 100.3 |
|  | WT (Ler) |  | ≤ 0.001 | F_2/384_ = 499.6 |
|  | *htl-2* |  | ≤ 0.05 | F_2/391_ = 3.2 |
|  | #18 |  | ≤ 0.001 | F_2/383_ = 104.8 |
|  | #23 |  | ≤ 0.001 | F_2/253_ = 127 |
| Fig. 3D | WT (Col) | Tukey | ≤ 0.001 | F_2/415_ = 1008 |
|  | *d14-1 kai2-2* |  | = 0.22 | F_2/353_ = 1.54 |
|  | *LjKAI2a #32* |  | ≤ 0.001 | F_2/287_ = 50 |
|  | *LjKAI2a #46* |  | ≤ 0.001 | F_2/184_ = 85 |
|  | *LjKAI2b #29* |  | ≤ 0.001 | F_2/283_ = 9.4 |
|  | *LjKAI2b #31* |  | ≤ 0.05 | F_2/244_ = 3.9 |
| Fig. 4B | LjKAI2a | Dunnett | ≤ 0.0001 | F_5/12_ = 96.1 |
|  | LjKAI2a^M160,L190^ |  | ≤ 0.001 | F_5/12_ = 9.5 |
|  | LjKAI2a^M160,L190, W157^ |  | = 0.227 | F_5/12_ = 1.63 |
|  | LjKAI2a ^W157^ |  | ≤ 0.05 | F_5/12_ = 4.17 |
|  | LjKAI2b |  | = 0.632 | F_5/12_ = 0.70 |
|  | LjKAI2b^L161,M191^ |  | = 0.001 | F_5/12_ = 8.9 |
|  | LjKAI2b ^L161,M191,F158^ |  | ≤ 0.0001 | F_5/12_ = 56.9 |
|  | LjKAI2b^F158^ |  | ≤ 0.0001 | F_5/12_ = 29.54 |
| Fig. 6C | - | Tukey | ≤ 0.001 | F_6/103_ = 35 |
| Fig. 6D | - | Tukey | ≤ 0.001 | F_4/67_ = 19.9 |
| Fig. 6E | - | Tukey | ≤ 0.001 | F_6/605_ = 26.5 |
| Fig. 7A | KAR1 PRL | Tukey | ≤ 0.001 | F_3/209_ = 7.40 |
|  | KAR1 PER |  | ≤ 0.001 | F_3/209_ = 11.1 |
|  | KAR1 PER density |  | ≤ 0.01 | F_3/209_ = 5.51 |
|  | KAR2 PRL |  | = 0.51 | F_3/217_ = 0.77 |
|  | KAR2 PER |  | = 0.18 | F_3/217_ = 1.64 |
|  | KAR2 PER density |  | = 0.72 | F_3/217_ = 0.44 |
|  | *rac*-GR24 PRL |  | = 0.74 | F_3/203_ = 0.42 |
|  | *rac*-GR24 PER |  | = 0.07 | F_3/203_ = 2.45 |
|  | *rac*-GR24 PER density |  | = 0.43 | F_3/203_ = 0.92 |
| Fig. 7B | - | Dunnett | ≤ 0.01 | F_3/188_ = 4.08 |
| Fig. 7C | WT | Tukey | ≤ 0.001 | F_2/9_ = 30.7 |
|  | *max2-4* |  | = 0.20 | F_2/9_ = 1.97 |
| Fig. 8A | - | Tukey | ≤ 0.001 | F_4/300_ = 8.69 |
| Fig. S8A | *KAI2a* | Tukey | ≤ 0.001 | F_5/18_ = 39.5 |
|  | *KAI2b* |  | ≤ 0.001 | F_5/18_ = 33.7 |
| Fig. S9D | - | Tukey | ≤ 0.001 | F_9/714_ = 178.8 |
| Fig. S10B | KAR1 | Tukey | ≤ 0.001 | F_3/396_ = 33.1 |
|  | KAR2 |  | ≤ 0.001 | F_3/390_ = 16.5 |
|  | *rac*-Gr24 |  | ≤ 0.001 | F_3/392_ = 35 |
| Fig. S10C | WT | Dunnett | ≤ 0.001 | F_2/313_ = 30 |
|  | *kai2a-1* |  | = 0.08 | F_2/234_ = 2.51 |
|  | *kai2b-1* |  | ≤ 0.001 | F_2/302_ = 29.3 |
|  | *kai2b-3* |  | ≤ 0.001 | F_2/308_ = 14.2 |
|  | *kai2a-1 kai2b-1* |  | = 0.99 | F_2/272_ = 0.01 |
| Fig. S10D | WT | Dunnett | ≤ 0.001 | F_2/246_ = 51 |
|  | *max2-4* |  | = 0.25 | F_2/204_ = 1.38 |
| Fig. S10E | WT | Dunnett | ≤ 0.001 | F_3/8_ = 28.4 |
|  | *kai2a-1* |  | ≤ 0.001 | F_3/8_ = 53 |
|  | *kai2b-3* |  | ≤ 0.001 | F_3/8_ = 26 |
|  | *kai2a-1 kai2b-1* |  | ≤ 0.001 | F_3/8_ = 105.8 |
|  | *max2-4* |  | = 0.99 | F_3/8_ = 0.04 |
